# Exploration of a Novel Catalytic Approach for Synthesizing Glycolide and ε-Caprolactone Copolymers and Their Application as Carriers for Paclitaxel

**DOI:** 10.3390/molecules30112318

**Published:** 2025-05-25

**Authors:** Rafał Wyrębiak, Ramona Figat, Ewa Oledzka, Adam Kasiński, Karolina Kędra, Anna Laskowska, Marcin Sobczak

**Affiliations:** 1 Department of Pharmaceutical Chemistry and Biomaterials, Faculty of Pharmacy, Medical University of Warsaw, Banacha 1 Str., 02-097 Warsaw, Poland; rafal.wyrebiak@wum.edu.pl (R.W.); eoledzka@wum.edu.pl (E.O.); adam.kasinski@wum.edu.pl (A.K.); 2 Department of Toxicology and Food Science, Faculty of Pharmacy, Medical University of Warsaw, Banacha 1 Str., 02-097 Warsaw, Poland; rfigat@wum.edu.pl; 3 Institute of Physical Chemistry, Polish Academy of Sciences, Kasprzaka 44/52 Str., 01-224 Warsaw, Poland; kkedra@ichf.edu.pl; 4 Department of Pharmaceutical Microbiology and Bioanalysis, Centre for Preclinical Research, Medical University of Warsaw, Banacha 1b Str., 02-097 Warsaw, Poland; anna.laskowska@wum.edu.pl

**Keywords:** biomaterials, biomedical polyesters, ε-caprolactone and glycolide copolymers, anti-cancer drug delivery systems, paclitaxel

## Abstract

Biodegradable polyesters serve as matrices in pharmaceutical applications for the controlled release of therapeutic agents. These polymers are essential in the advancement of drug delivery systems (DDSs) that facilitate the gradual drug release over a predetermined duration. Therefore, this study introduces the novel use of a diethyl zinc/propyl gallate catalytic system to synthesize glycolide/ε-caprolactone copolymers (PGCL) for subsequent biomedical applications. A total of twenty-four biodegradable copolymeric matrices, characterized by a highly random microstructure and an average molecular weight (*M*_n_) ranging from approximately 27 to 62 kDa, were synthesized and analyzed. The resulting copolymer samples underwent Neutral Red Uptake (NRU) and Umu tests, revealing no signs of cyto- or genotoxicity. Furthermore, a hemolysis assay was conducted on selected samples, indicating their suitability for intravenous administration. Finally, a release study of paclitaxel (PACL) from one of the synthesized matrices demonstrated a sustained and highly controlled drug release profile, following first-order kinetics and the Fickian diffusion mechanism.

## 1. Introduction

There is an increasing focus on catalytic systems that employ non-toxic metals. The inherent safety of these materials ensures that the resulting polymers are suitable for medical applications, thereby significantly reducing the likelihood of adverse reactions when they interact with biological systems. This safety is particularly critical in fields such as drug delivery and tissue engineering. Furthermore, these catalysts align with the principles of green chemistry, which prioritize minimizing environmental impact. By reducing the reliance on hazardous substances and generating fewer toxic byproducts, non-toxic metal catalysts advocate for a more sustainable and environmentally conscious approach to polymer synthesis. Another notable benefit of these catalysts is their high efficiency; they frequently exhibit remarkable efficiency and selectivity, leading to enhanced yields and purer products. This aspect is especially vital in the biomedical sector, where the quality and purity of materials can directly affect their efficacy and safety. The demand for these catalytic systems is also influenced by sustainability concerns. The adoption of non-toxic metals fosters the creation of sustainable materials, which is crucial for long-term environmental well-being and responsible resource management. This approach is in harmony with broader objectives aimed at reducing our ecological footprint and promoting sustainable practices across various scientific and technological domains.

Biodegradable polyesters are widely used and investigated as biomaterials (e.g., sutures, drug delivery systems (DDSs)), biodegradable food packaging and coatings [1,2,3,4,5]. The production of these materials is predominantly achieved via two principal techniques: the polycondensation of hydroxyacids and the ring-opening polymerization (ROP) of cyclic lactones. Of the two, ROP is the preferred method due to its more controllable reaction, higher purity, and higher yield of the final products. Despite the numerous different catalysts of ROP that have been developed over the years, tin (II) octanoate (Sn(Oct)_2_) still remains the most used catalyst. However, tin residues can pose a potential health threat and environmental concerns [6,7], and the catalyst is classified as potentially damaging to an unborn child. It also requires moderately high temperatures (ca. 100 °C) to achieve satisfactory monomer conversion in a reasonable time [8,9,10].

The most used cyclic monomers for ROP of biomedical polymers are ε-caprolactone (CL), lactide (LA), glycolide (GL), and trimethylene carbonate (TMC). The biodegradation rate of a biomedical polymer depends on the polymer mass, its microstructure (randomness, monomer chain length), tacticity (for polymers synthesized from chiral monomers), and monomer composition. Modifying the specified characteristics could enable the development of biomedical polymers with a targeted degradation time. Various copolymers of the aforementioned cyclic monomers were also developed and are mainly used as materials for resorbable sutures. Examples of such copolymers are Monosyn (GL–co–CL–co–TMC), Caprosyn (GL–co–L–LA–co–CL–co–TMC), Monocryl (GL–co–CL), Maxon (GL–co–TMC) [11]. Copolymerization of these cyclic monomers yields copolymers with properties intermediate to homopolymers of respective monomers [12]. Numerous catalysts for ROP of cyclic lactones have been developed over the years; the main groups are enzymatic, coordinating, and ionic [13,14,15]. Notable examples are alkali metal alkoxides [15,16], strontium and aluminum isopropoxide [17,18], zirconium acetylacetonate [19], neodymium [20], gallium [21,22], and titanium [23] complexes.

As complete removal of catalyst residues from the final polymer can be impractical or very challenging, some of the catalysts are especially interesting due to their low toxicity and biocompatibility. Physiologically occurring or biologically inert elements are preferred. Examples include zirconium acetylacetonate [19], zinc undecylenate [7], zinc and copper salts of phenylalanine [24], iron complexes of guanidine [25], or numerous calcium complexes [26].

Poly(ε-caprolactone) (PCL) is characterized by high biocompatibility and favorable mechanical properties, but its in vivo degradation is very slow (even several months). Polyglycolide (PGA) degrades rapidly (up to ca. 6 months), but its application in DDSs is limited due to the low solubility of homopolymer. Polylactide (PLA), however, degrades significantly faster (even within a few weeks, depending on microstructure) [27,28,29,30].

Numerous therapeutic systems featuring prolonged-release formulations that utilize polyesters are currently available in the market. Leuprostin is a prefilled syringe with an implant containing 3.6 mg of leuprorelin acetate incorporated in poly(lactic-*co*-glycolic acid) (PLGA). A cylindrical, 10 mm long rod is injected subcutaneously into the anterior abdominal wall and releases active ingredients for 1 month. It is indicated in the treatment of hormone-dependent prostate cancer [31]. Reseligo is an example of a similar product. It is a subcutaneous implant containing 3.6 mg of goserelin acetate incorporated in a 50:50 poly(D,L-lactide-*co*-glycolide) copolymer. Indications include prostate cancer, breast cancer, and endometriosis. The implant should be administered every 28 days [32]. Rispolept Consta is a suspension containing 25, 37.5, or 50 mg of risperidone incorporated in poly(D,L-lactide-co-glycolide) copolymer as well. After intramuscular injection, less than 1% of the drug was released immediately. The main release occurs after a 3-week latency period, from the fourth to the seventh week. It is indicated in the maintenance therapy of schizophrenia [33].

PGCL copolymers are the subject of limited research as drug carriers. Jaworska et al. used a PGCL-PLGA electrospun blend as an implantable paclitaxel DDS in a mouse model of breast cancer [34]. The same team developed papaverine–loaded PGCL nonwovens with potential urological applications [35]. Zhang et al. made functionalized, ovalbumin–loaded PGCL microspheres incorporated in temperature–sensitive hydrogel [36].

In terms of long–term stability, PGCL generally degrades faster than PCL. However, the degradation rate highly depends on molecular mass [37]. Like PCL, PGCL’s crystallinity increases during hydrolytic degradation, and amorphous regions of the polymer are, therefore, more prone to hydrolysis.

In comparison to PLGA, PGCL degrades more slowly [35]. Due to rapid degradation, PLGA is used in commercial prolonged-release medications, as mentioned above. It is worth noting, however, that degradation products of PLGA (lactic acid and glycolic acid) are highly acidic. Furthermore, higher local acidity increases the degradation rate, leading to an auto-catalytic process and may cause toxicity or inflammation locally due to low pH value [38]. On the contrary, the main product of low GL content PGCL degradation—6-hydroxyhexanoic acid—is significantly weaker than lactic or glycolic acid. This should minimize the risk of low pH-induced local adverse effects.

Type-II transesterification is a phenomenon where a polymer chain is attacked by another actively growing polymer chain-catalyst complex. This may result in the cleavage of a glicolidyl unit and the formation of abnormal sequence caproyl–glycoyl–caproyl units, which can only form when the type-II transesterification process is present. The occurrence of these sequences increases the randomness of the copolymer chain, decreasing crystallinity. As mentioned before, decreased crystallinity generally lowers the hydrolytic resistance of the polymer [9,34].

Paclitaxel (PACL) is a naturally occurring chemotherapeutic agent isolated from several species of yew trees. It prevents microtubule depolymerization and promotes the microtubule assembly from tubulin dimmers. It is used in the treatment of several cancers, including breast cancer, ovarian cancer, adenocarcinoma, non-small cell lung cancer, and AIDS-related Kaposi’s sarcoma. Available formulations include concentrate for solution for infusion (containing macrogolglycerol ricinoleate and ethanol) and albumin-bound nanoparticles [39,40].

In our previous works, a novel diethylzinc/propyl gallate catalytic system was successfully used as an ROP initiator for copolymerization [41,42] of cyclic lactones. The resulting polymers were found to be neither cytotoxic nor genotoxic. The scope of the current study is to evaluate the aforementioned catalytic system’s activity as the catalyst for the copolymerization of CL and GL in order to obtain biodegradable copolymeric matrices for PACL delivery. As far as we are aware, there are no documented instances in the literature that describe the application of this catalytic system in the synthesis of poly (ε-caprolactone-*co*-glycolide) copolymers (PGCL).

## 2. Results and Discussion

### 2.1. Synthesis and Characterization of PGCL Copolymers

**Figure 1:**
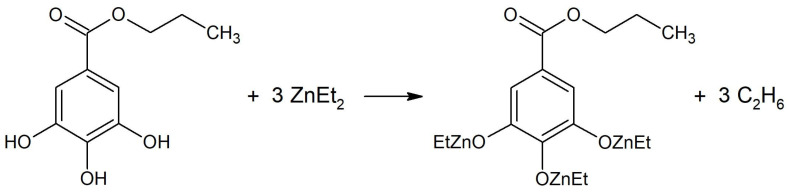
Simplified synthesis of the catalytic system.

The catalytic system was prepared as in our previous work, where it was used for copolymerizing CL and LA [42]. In short, the FDA-approved food additive propyl gallate was reacted with three equivalents of diethylzinc at 0 °C (Figure 1). Despite multiple efforts, the crystallization and structural analysis of the catalytic system were unsuccessful. We propose that the lack of crystallinity in the system’s structure is attributable to the formation of macromolecular polyphenolic gallate associates bridged by zinc cations.

**Figure 2:**
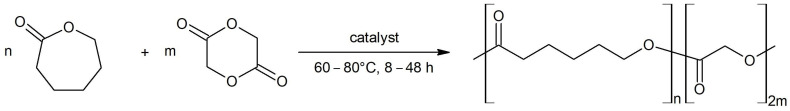
Schematic outline for the synthesis of PGCL copolymers.

In the first step of the investigation, twenty-four matrices of PGCL were successfully synthesized using the ROP process (Figure 2, Table 1). The loading of GL monomer was maintained at a low level to prevent the formation of insoluble polymeric products. A range of reaction conditions, including time, temperature, catalyst concentration, and comonomer ratio, were employed. The resulting polymers were characterized by a high molecular mass (*M*_n_) (ca. 28–63 kDa) and high randomness (~100%). Complete conversion of GL was observed across all entries, with the absence of GL monomer evident in the ^1^H NMR spectra of the raw mixture following the reaction. Similarly, CL conversion was nearly complete in most instances. The dispersity values exhibited a range from moderately low to moderately high (1.49 to 2.99). Type-II transesterification ratios ranged from a moderate 48% to a complete 100%, depending on the specific entry. It is reasonable to deduce that the low loading of GL monomers resulted in medium lengths of CL chains being considerably longer, generally between 6 and 10 units, in contrast to the medium lengths of GL chains (0.57 to ca. 1 unit).

In comparison to the widely used Sn(Oct)_2_, our catalytic system yielded higher CL conversion both at 60 °C (over 96% after 24 h vs. 65% after 96 h for Sn(Oct)_2_) and at 80 °C (over 96% after 16 h vs. 62% after the same time for Sn(Oct)_2_). The resulting polymers were also characterized by significantly higher molecular mass (*M*_n_ = ca. 6–10 kDa for Sn(Oct)_2_) [8].

### 2.2. Differential Scanning Calorimetry

**Figure 3:**
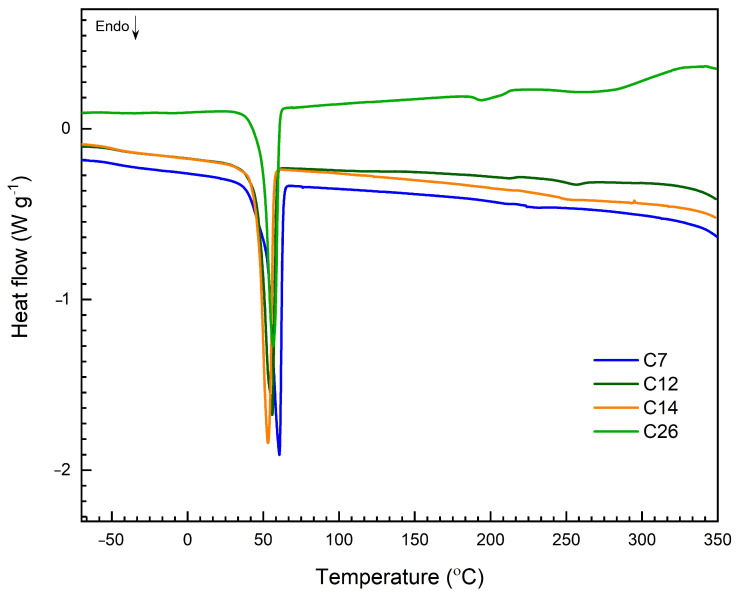
DSC curves of the selected copolymers.

The thermal properties of the copolymeric samples were determined by Differential Scanning Calorimetry (DSC) (Figure 3). The DSC curves follow a similar pattern with one melting peak indicating melting points (T_m_, onset) between 43 and 51 °C derived from PCL units (Table 2). No indications of a glass transition (T_g_) of copolymers were found. Furthermore, the T_m_ associated with GL sequences was absent, which can be attributed to the limited quantity of GL units present in the copolymer chain, corroborating earlier published research [43,44]. Another factor contributing to the lack of a specific T_m_ for GL units in DSC analysis is their tendency to generate amorphous regions within the copolymer matrix, which do not possess a well-defined crystalline T_m_ [45].

### 2.3. Toxicity Studies

The selected copolymer samples were evaluated for cyto- and genotoxicity (Table 3). All samples were non-toxic according to the Neutral Red Uptake (NRU) test; the cell viability reduction was less than 30%). The developed catalytic system also seems to be very promising from the point of view of biological safety. Possible toxicity could be related to post-catalytic residues in the polymerization products. As is known, the FDA recognizes propyl gallate as being generally safe.

### 2.4. Hemolysis Assay

**Figure 4:**
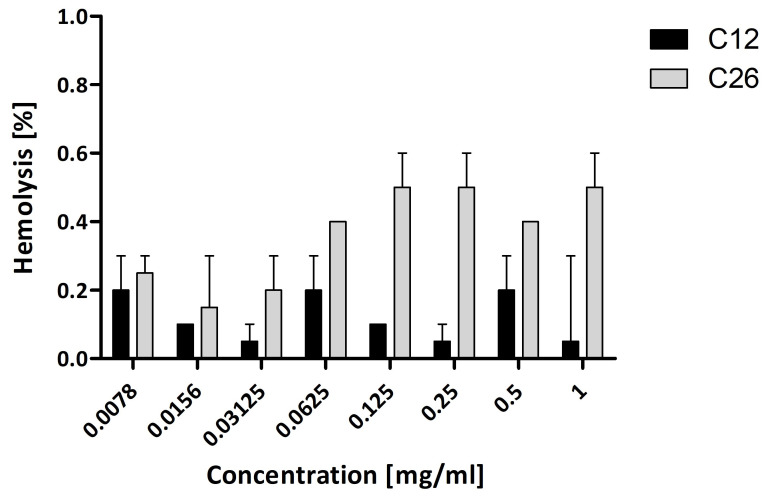
Hemolysis induced by tested compounds after 1 h of incubation.

The hemolysis assay is frequently employed to assess the toxicity of various agents under investigation. In the case of the synthesized biodegradable matrices, the observed hemolysis levels were minimal (refer to Figure 4). For both copolymeric materials examined, the hemolysis did not surpass 1%, irrespective of concentration. According to the ASTM F756 Standard [46], material is considered non-hemolytic if the level of hemolysis it induces is between 0 and 2%. Furthermore, no statistically significant differences were identified among the compounds. These findings suggest that the matrices tested can be administered intravenously without safety concerns [47].

### 2.5. PACL Release Kinetics Studies

**Figure 5:**
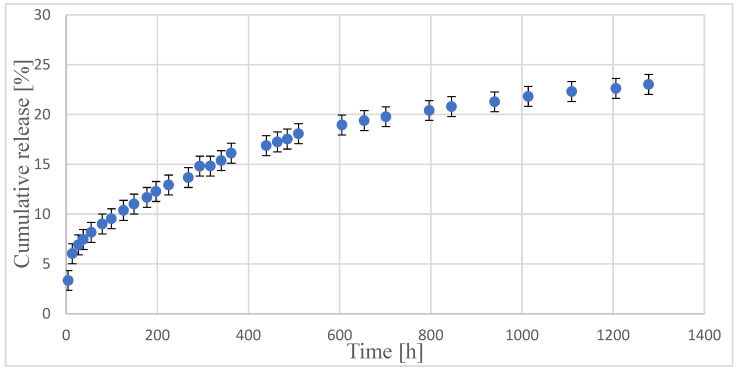
In vitro PACL released profile.

An in vitro investigation was conducted to assess the release of PACL from the model copolymeric carrier at a pH of 7.4 and a temperature of 37 °C over a duration of 53 days. The ordinate of the plot was calculated based on the cumulative amount of drug released concerning its initial amount in the polymer. The kinetics release PACL from the obtained polymeric carrier was investigated (Figure 5).

The data points derived from the PACL release studies underwent analysis using zero-order and first-order kinetics, as well as the Higuchi and Korsmeyer-Peppas models, to investigate the kinetics and mechanisms of drug release from the copolymeric carrier (Table 4). As is known from the literature, according to the Korsmeyer-Peppas model, for the diffusion-degradation controlled DDSs, the release exponent value *n* is in the range of 0.45 and 0.89 (anomalous, non-Fickian). In contrast, when *n* is close to 0.45, the diffusion (Fickian diffusion) predominates in the process, and, in the opposite case, *n* > 0.89, the model corresponds to the super case II transport [31].

It was noted that PACL was released from a polymeric carrier with rather first-order kinetics (R^2^ was 0.852). According to the drug release mechanism considerations, the examined profile matches the Higuchi model perfectly, implying a diffusional release mechanism. Furthermore, the analysis of PACL release data using the Korsmeyer-Peppas model suggested that the PACL release from the polymeric carrier was governed instead by a Fickian transport (*n* = 0.329).

As reported in the literature [48], highly random PGCL copolymers with low GL content are relatively stable hydrolytically. Their weight loss is less than 20% after 26 weeks of incubation at 37 °C at pH = 7.4. This strengthens the diffusional release mechanism further.

## 3. Materials and Methods

### 3.1. Chemicals

Glycolide (1,4-dioxane-2,5-dione, purity ≥ 99%, GL), toluene (anhydrous, purity 99.8%), propyl gallate (purity ≥ 98%), Cremophor^®^ EL and diethylzinc solution (15% wt. in toluene) were purchased from Sigma-Aldrich (Poznań, Poland) and used as received. ε-caprolactone (2-oxepanone, purity 97%, CL) was purchased from Sigma-Aldrich (Poznań, Poland) and distilled over calcium hydride before use. Dichloromethane (pure, 99%) and methanol (pure, 99.9%) were purchased from Chempur (Piekary Śląskie, Poland) and distilled before use. Concentrated hydrochloric acid (35–38%, pure for analysis) was purchased from Chempur (Piekary Śląskie, Poland) and used as received.

### 3.2. Polymerization Procedure

Copolymerization was performed analogously, as in our previous work [42].

GL (91 mg) was placed in a dry, 10 mL reaction vessel. The vacuum was applied for ca. 10 min; then, the reaction vessel was evacuated and backfilled with argon 3 times. 1 mL of CL and 3 mL of dry toluene were added through a gas adapter with a dry argon flow. The reaction vessel was sealed and heated gently while stirring until full GL dissolution. 1 mL of catalyst suspension (containing 98 μmol of zinc) was added, and the reaction vessel was resealed, stirred vigorously, and placed in a preheated (80 °C) oil bath for 16 h. After that, the post-reaction mixture was removed from the bath, cooled, and diluted in 10 mL of CHCl_3_. The copolymer was precipitated by adding CHCl_3_ solution to a freezing-cold (ca. −20 °C) concentrated hydrochloric acid-methanol (1:9 *v*:*v*) mixture. To further purify the copolymer, it was additionally redissolved in 10 mL of CHCl_3_ and reprecipitated from ice-cold methanol 2 more times.

### 3.3. PACL Delivery System Preparation

90 mg of copolymer C22 was placed in a 10 mL vial and suspended in 1 mL of CHCl_3_ with vigorous stirring. 10 mg of PACL in 3 mL of CHCl_3_ was added. The resulting thick solution was gently stirred for 10 min. The solution evaporated overnight to yield the delivery system as a thin polymer film. Due to the procedure, quantitative drug incorporation in the delivery system was assumed.

### 3.4. PACL Release Kinetics Studies

99.1 mg of the PACL delivery system was placed in a 5 mL vial covered in aluminum foil. 4 mL of phosphate buffer (pH = 7.4) with 1% Cremophor^®^ EL was added. The vessel was placed in an ES-20 Grant-bio orbital shaker-incubator (incubation temperature 37 °C, 100 rpm). After a predetermined time, 1 mL of the release medium was withdrawn and replaced with 1 mL of fresh, preheated buffer.

The release data points were subjected to zero-order, first-order kinetics, Higuchi, and Korsmeyer–Peppas models, respectively. Calculations were made based on the formulas mentioned below [49]:

(1) Zero-order: F=kt

(2) $$\mathrm{First}\text{-}\mathrm{order}:\;\log F=\log F_{0}-\frac{kt}{2.303}$$

(3) $$\mathrm{Higuchi}\;\mathrm{model}:\;\log F=k\sqrt{t}$$

(4) $$\mathrm{Korsmeyer}\text{-}\mathrm{Peppas}\;\mathrm{model}:\;F=kt^{n}\;(F<0.6)$$

where

*F* is the fraction of drug released from the matrix after time *t*;

*F*_0_ is the initial amount of drug,

*k* is a model constant, and n is the drug release exponent in the Korsmeyer–Peppas model.

### 3.5. HPLC Measurements

The analyses were performed using Dionex apparatus, with HPLC pump model 7580, DAD detector UVD 340S (Dionex, Sunnyvale, CA, USA), and Jetstream II Plus (WO Industrial Electronics, Vienna, Austria) thermostat. Phenomenex Luna C-18 HPLC column (25 cm × 4.6 mm; particle size 5 μm) (Phenomenex, Torrance, CA, USA), combined with Phenomenex C-18 (4 mm × 3 mm) pre-column (Phenomenex, Torrance, CA, USA) was used as a stationary phase. A mixture of acetonitrile: methanol: water (60:2:38; *v*/*v*) with a 0.1% addition of trifluoroacetic acid was used as a mobile phase. All samples were pre-filtered using a 0.22 μm syringe filter. The method was validated previously [50].

### 3.6. Toxicity Studies

Cytotoxicity of the polymeric materials was evaluated according to the ISO 10993-5:2009 guideline [51] by the Neutral Red Uptake (NRU) test using BALB/c T3T clone A31 mice fibroblasts cell line (American Type Culture Collection). The polymeric extracts were prepared by incubating the samples in 1 mg/mL DMEM medium with 10% bovine serum for 24 h at 37 °C with stirring; the extracts were sterilized on a syringe filter. Highly cytotoxic latex and non-cytotoxic polyethylene were used as reference materials. Samples were considered cytotoxic if they reduced cell survival below 70% compared to the untreated cells.

Genotoxicity of the polymeric materials was evaluated by the Umu test using *Salmonella typhimurium* TA3515/psk1002 (Deutsche Sammlung von Mikroorganismen und Zellkulturen GmbH, Braunschweig, Germany) under the ISO 13829:2000 guideline [52]. The experiment was conducted with and without metabolic activation (S9 rat liver fraction). The polymeric extracts were prepared by incubating the samples in 1 mg/mL PBS buffer (GIBCO) for 24 h at 37 °C. All of the extracts were sterilized by filtration before the assay. The 2-aminoanthracene and 4-nitroquinoline N-oxide were used as positive controls. The bacteria growth factor (G) is evaluated to determine cytotoxicity, and the Induction Ratio (IR) is to determine the genotoxic potential of tested samples. Samples were considered genotoxic when the IR value was ≥1.5.

### 3.7. Hemolysis Assay

Sheep Blood Defibrinated was obtained from Biomaxima (Lublin, Poland). The blood was centrifuged for 10 min at 2000 rpm. Plasma was gently removed, and PBS (pH 7.4, RT) was added. The solution was mixed gently and centrifuged for 10 min at 2500 rpm. The washing step was repeated three times. After the last washing, the supernatant was removed, replaced with fresh PBS (pH 7.4, RT), and gently mixed. RBCs were diluted in PBS (pH 7.4) to obtain a 10% and 2% suspension of RBC. 2% RBC suspension was then incubated with increasing concentration of the compounds in a 1:1 ratio for 1 h at 37 °C. Next, samples were centrifuged at 4000 rpm for 5 min, and 100 μL of supernatant from each sample was transferred to a 96-well plate. Optical density (OD) was measured at 540 nm. Sterile, distilled water, and PBS were used to prepare positive and negative controls, respectively. The experiment was performed in triplicate. The value of nanoparticle-induced hemolysis was calculated (Equation (5)):

(5) $$\mathrm{Hemolysis}\;\left[\%\right]=\frac{A-A0\%}{A100\%-A0\%}\times 100\%$$

where A—absorbance of the sample, A100%—absorbance of positive control (100% hemolysis), A0%—absorbance of negative control (0% hemolysis).

Statistical analysis for hemolysis results was performed using Graph Pad Prism 5 software. Kruskal–Wallis test with Dunn post-test was used to show the dependence between hemolysis level and matrix concentration. Comparison of hemolysis level between two matrices was performed using Two-way ANOVA with Bonferroni post-test.

### 3.8. GPC Measurements

Relative average molecular mass and molecular mass distribution were determined by Gel Permeation Chromatography (GPC). GPC instrument (GPC Max + TDA 305, Viscotek, Malvern, UK) was equipped with Jordi DVB Mixed Bed columns (one guard and two analytical) at 30 °C in CH_2_Cl_2_ (HPLC grade, Sigma-Aldrich, Poznań, Poland), at a flow rate of 1 mL/min with RI detection and calibration based on narrow PS standards (ReadyCal Set, Fluka, Poznan, Poland). The results were processed with OmniSEC software (version 5.12). *M*_n_ values were not corrected.

### 3.9. NMR Measurements

All the spectra were recorded on an Agilent Technologies 400 MHz (Santa Clara, CA, USA) spectrometer. Chloroform-d (CDCl_3_, Sigma-Aldrich) with TMS internal standard (0.1% *v*/*v*) was used as a solvent. Typical chemical shifts values are included in Table 5 and Table 6. Sample ^1^H (Figure S1) and ^13^C (Figure S2) spectra are available in Supplementary Materials.

Microstructures of copolymers were analyzed as reported in the literature [9]. In short, measured average lengths of glycolyl (*L_G_*) and caproyl (*L_C_*) blocks were calculated as follows:

*L*_G_ = *L*_C_/*k* (6)

*L*_C_ = ([CC] + [GC])/[GC] (7)

where [CC] and [GC] are the concentrations of the respective signals in the NMR spectrum, and

*k* = [C]/[G] (8)

where [C] and [G] are the experimental concentrations of caproyl and glycolyl units in copolymer.

The second mode transesterification coefficient is calculated from Equation (9):

*T*_II_ = [CGC]/[CGC]_R_ (9)

where [CGC] is the measured concentration of transesterified CGC sequence and [CGC]_R_ is the calculated concentration of the aforementioned sequence for a completely random chain:

[CGC]_R_ = *k*^2^/(*k* + 1)^3^ (10)

The degree of randomness (*R*) was calculated as follows:

*R* = *L*_G_^R^/*L*_G_ = *L*_C_^R^/*L*_C_ (11)

where *L_G_^R^* and *L_C_^R^* are the lengths of glycolyl and caproyl blocks in the completely random copolymer chains, calculated as follows:

*L*_C_^R^ = *k* + 1 (12)

*L*_G_^R^ = (*k* + 1)/*k* (13)

### 3.10. DSC Measurements

All the measurements were recorded using a DSC 3 Mettler Toledo device. A 3–7 mg sample of an analyzed compound was placed in an aluminum crucible closed with a hole lid and tested in heating-cooling cycles. First, the samples were cooled to −80 °C and equilibrated for 15 min. After that, the DSC curve was recorded during heating to 250 °C and cooling back to −80 °C at a 5°/min rate. Then, after 10 min isothermal step at −80 °C, the heating step was recorded at a rate of 20°/min. All measurements were carried out under a nitrogen atmosphere.

## 4. Conclusions

In summary, CL and GL copolymers were synthesized using a novel diethylzinc/propyl gallate catalytic system. Copolymers were characterized by varying microstructure (L_C_, L_GG_, T^II^ ratio). As all tested copolymers were found to be non-hemolytic, cyto- or genotoxic, it can be presumed that the tested catalytic system is suitable for the synthesis of biomedical polymers. Based on this assumption, the PACL DDS prototype was prepared, and it was found to release PACL with first-order kinetics, according to Fickian diffusion transport. As 23% of PACL was released from the DDS within 53 days, it could be classified as a long–term DDS. We hope that the obtained PACL carrier will find application in the future in the technology of DDSs characterized by high control of the release of anti-cancer drugs.

## Figures and Tables

**Table 1:** Copolymerization of GL and CL Characterization of the synthesized copolymeric matrices.

Entry	Reaction Time [h]	Reaction Temperature [°C]	Molar Ratio Zn/Monomers [%]	Molar Ratio GL/CL [%]	CL Conversion [%]	*Đ* ^a^	*T*^II b^ [%]	*L*_C_ ^c^	*L*_GG_ ^d^	*M*_n_ ^e^ [kDa]	*R* ^f^ [%]
C1	8	80	1.0	8.0	89	1.92	99	8.18	0.59	45.0	100
C2	16	80	1.0	8.0	98	2.05	84	8.93	0.64	43.2	100
C3	24	80	1.0	8.0	97	1.48	97	7.87	0.59	31.8	100
C4	48	80	1.0	8.0	99	1.84	92	7.92	0.61	41.5	100
C5	8	80	2.0	8.0	97	1.92	100	9.42	0.57	38.9	100
C6	16	80	2.0	8.0	98	1.73	90	10.18	0.61	48.2	100
C7	24	80	2.0	8.0	99	1.85	86	10.30	0.63	42.5	100
C8	48	80	2.0	8.0	99	1.97	88	10.53	0.62	36.4	98
C9	8	80	1.0	15.0	86	1.56	100	3.74	0.58	36.2	100
C10	16	80	1.0	15.0	96	2.20	100	6.11	0.59	38.5	100
C11	24	80	1.0	15.0	91	2.04	100	6.50	0.59	48.8	100
C12	48	80	1.0	15.0	99	1.56	100	5.92	0.61	62.9	100
C13	8	80	2.0	15.0	97	1.73	94	8.96	0.61	38.2	100
C14	16	80	2.0	15.0	98	1.76	100	5.83	0.59	35.1	100
C15	24	80	2.0	15.0	99	1.48	79	9.11	0.66	38.0	100
C16	48	80	2.0	15.0	99	1.78	80	7.96	0.68	41.2	100
C17	24	60	1.0	8.0	98	1.99	97	8.99	0.61	32.9	99
C18	48	60	1.0	8.0	98	1.79	100	1.91	0.66	43.0	100
C19	24	60	2.0	8.0	98	1.67	90	9.40	0.64	42.1	100
C20	48	60	2.0	8.0	98	1.71	77	10.36	0.67	27.7	100
C21	24	60	1.0	15.0	96	2.99	57	7.63	1.05	38.1	100
C22	48	60	1.0	15.0	96	1.84	48	17.14	0.85	42.2	100
C23	24	60	2.0	15.0	97	2.25	100	6.52	0.60	33.6	100
C24	48	60	2.0	15.0	97	1.74	100	6.64	0.60	39.8	100

^a^ Dispersity (*Đ*), ^b^ Type II transesterification, ^c^ Median length of caproyl units, ^d^ Median length of glycolidyl units, ^e^ Number average molar mass (*M*_n_), ^f^ randomness.

**Table 2:** Melting peaks characteristics of the selected copolymers.

Sample	*T*_m, onset_ (°C)	ΔH (J/g)
C7	43.1	−506.9
C12	50.7	−334.0
C14	47.1	−548.0
C18	47.1	−222.2

**Table 3:** Cytotoxicity results for the selected copolymers.

Sample	Genotoxicity Assay				Cytotoxicity Assay
	−S9 ^a^		+S9 ^b^		
	G ± SD	IR ± SD	G ± SD	IR ± SD	Cells Viability ± SD [%]
C7	1.10 ± 0.11	1.09 ± 0.07	1.05 ± 0.04	0.94 ± 0.06	103 ± 4
C12	1.16 ± 0.16	0.92 ± 0.27	1.09 ± 0.01	0.91 ± 0.08	93 ± 1
C14	1.14 ± 0.05	0.85 ± 0.13	1.08 ± 0.03	0.83 ± 0.14	116 ± 3
C18	1.07 ± 0.02	0.89 ± 0.12	1.11 ± 0.03	0.86 ± 0.14	113 ± 7
C20	1.01 ± 0.02	0.95 ± 0.12	1.01 ± 0.02	0.91 ± 0.14	114 ± 1
PC ^c^	1.04 ± 0.01	3.30 ± 0.28	0.87 ± 0.04	2.53 ± 0.41	1 ± 1
NC ^d^	1.00 ± 0.01	1.00 ± 0.05	1.00 ± 0.04	1.00 ± 0.08	111 ± 1

^a^ Version without metabolic activation, ^b^ Version with metabolic activation, ^c^ Positive control—2-aminoanthracene (*umu*-test with S9 fraction), 4-nitroquinoline N-oxide (*umu*-test without S9 fraction), latex (NRU test), ^d^ Negative control—deionized sterile water (*umu*-test), polyethylene film (NRU test).

**Table 4:** Analysis data of PACL release from obtained polymeric carrier.

Model Type	*R* ^2^	*n* (Transport Mechanism)
Zero-order model	0.831	-
First-order model	0.852	-
Higuchi model	0.969	-
Korsmeyer-Peppas model	0.993	0.329 (Fickian diffusion)

**Table 5:** Approximated ^1^H NMR chemical shifts in the synthesized copolymers.

Proton ^a^	δ [ppm]
–C(O)C**H_2_**O– (comonomeric)	4.85–4.68
–C(O)C**H_2_**O– (transesterified)	4.60
–C(O)CH_2_CH_2_CH_2_CH_2_C**H_2_**O– (comonomeric)	4.17–4.13
–C(O)CH_2_CH_2_CH_2_CH_2_C**H_2_**O– (homomonomeric)	4.06–4.03
–C(O)C**H_2_**CH_2_CH_2_CH_2_CH_2_O– (comonomeric)	2.45–2.40
–C(O)C**H_2_**CH_2_CH_2_CH_2_CH_2_O– (homomonomeric)	2.32–2.27
–C(O)CH_2_C**H_2_**CH_2_C**H_2_**CH_2_O–	1.71–1.60
–C(O)CH_2_CH_2_C**H_2_**CH_2_CH_2_O–	1.44–1.33

^a^ Chemical shits values refer to bolded protons.

**Table 6:** Approximated ^13^C NMR chemical shifts in the synthesized copolymers.

Carbon ^a^	δ [ppm]
–**C**(O)CH_2_CH_2_CH_2_CH_2_CH_2_O–	174.0–173.2
–**C**(O)CH_2_O–	168.0
–C(O)CH_2_CH_2_CH_2_CH_2_**C**H_2_O–	65.6–64.6
–C(O)**C**H_2_O–	61.0
–C(O)**C**H_2_CH_2_CH_2_CH_2_CH_2_O–	34.5–34.1
–C(O)CH_2_CH_2_CH_2_**C**H_2_CH_2_O–	28.8–28.6
–C(O)CH_2_CH_2_**C**H_2_CH_2_CH_2_O–	26.0–25.8
–C(O)CH_2_**C**H_2_CH_2_CH_2_CH_2_O–	25.0–24.9

^a^ Chemical shits values refer to bolded carbons.

## Data Availability

Data are contained within the article and Supplementary Materials.

## Supplementary Materials

The following supporting information can be downloaded at: https://www.mdpi.com/article/10.3390/molecules30112318/s1. Figure S1. ^1^H NMR spectrum of a synthesized PGCL copolymer (CDCl_3_). Figure S2. ^13^C NMR spectrum of a synthesized PGCL copolymer (CDCl_3_).
