# Supplementary material for: Exploration of a Novel Catalytic Approach for Synthesizing Glycolide and ε-Caprolactone Copolymers and Their Application as Carriers for Paclitaxel
Source: Molecules. 2025 May 25;30(11):2318. doi: 10.3390/molecules30112318 (PMC12156253; doi:10.3390/molecules30112318)
Supplement: Supplementary file 1 [file molecules-30-02318-s001.zip › molecules-3587857-supplementary.pdf]

Exploration of a novel catalytic approach for synthesizing glycolide and ε-caprolactone copolymers and their application as carriers for paclitaxel

Rafał Wyrębiak, Ramona Figat, Ewa Oledzka, Adam Kasiński , Karolina Kędra, Anna Laskowska and Marcin Sobczak

Supporting information

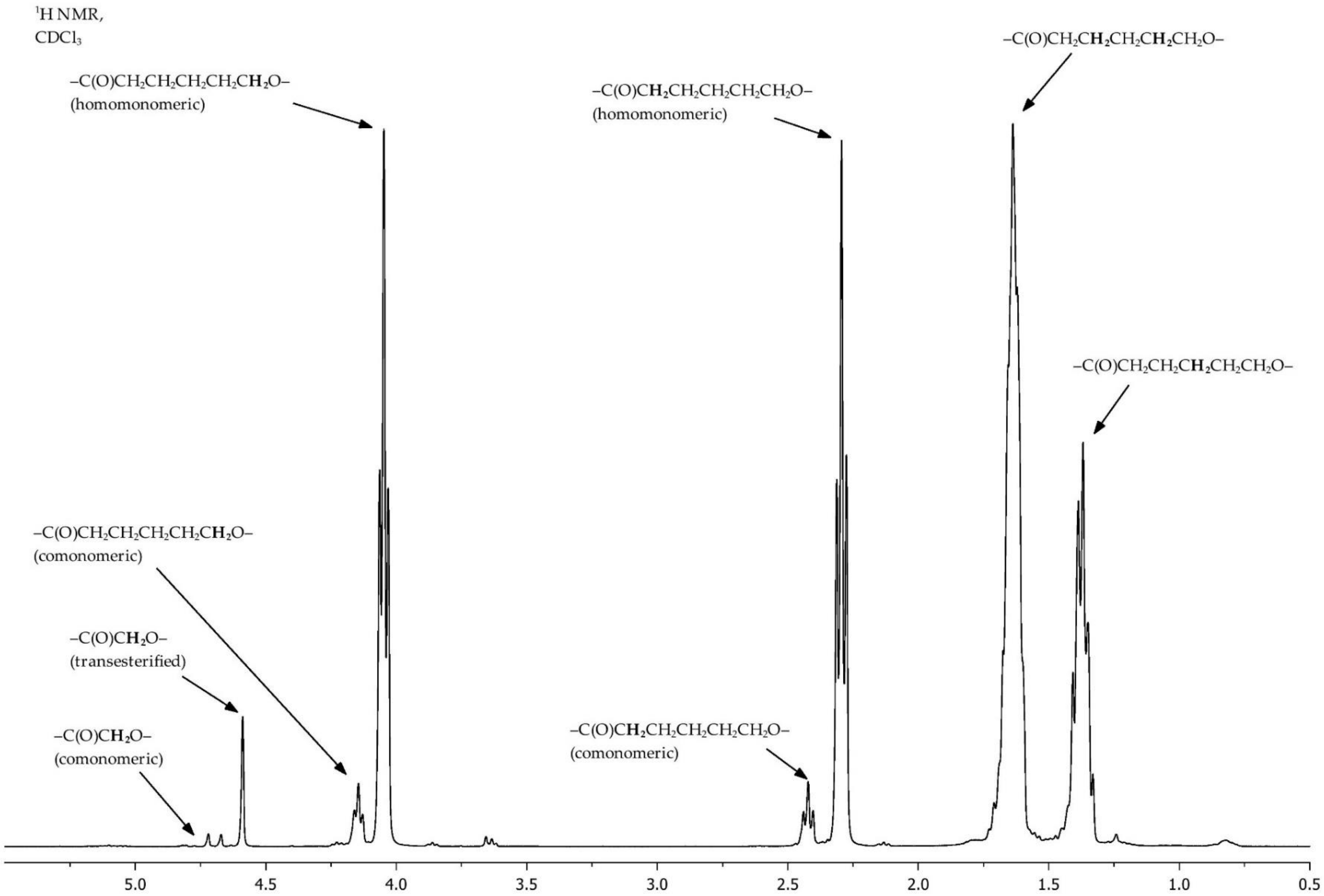

Figure S1. ^{1}H NMR spectrum of a synthesized PGCL copolymer ($CDCl_3$).

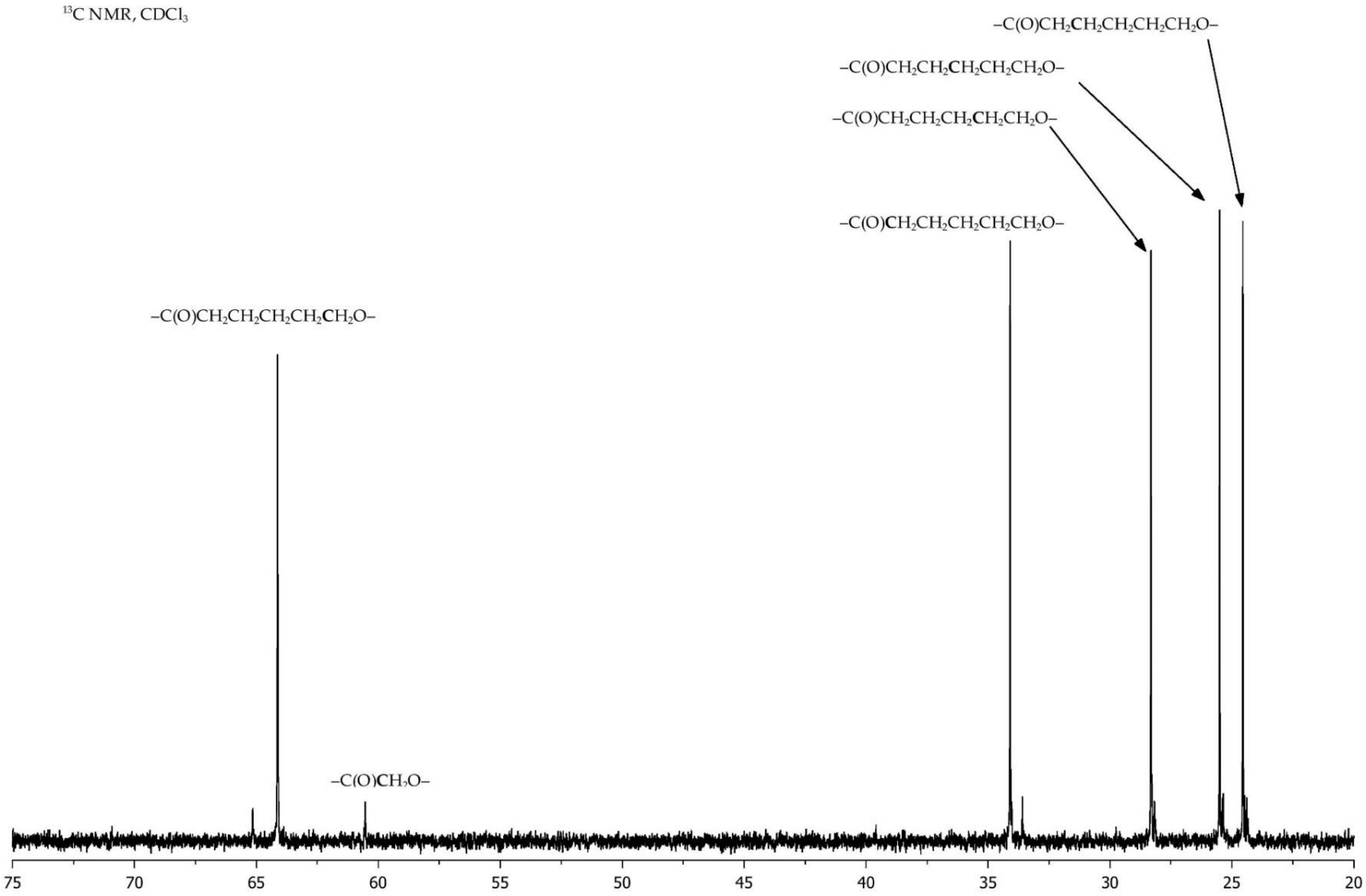

Figure S2. ^{13}C NMR spectrum of a synthesized PGCL copolymer ($CDCl_3$).
